# Integrated bioinformatic analysis of protein landscape in gingival crevicular fluid unveils sequential bioprocess in orthodontic tooth movement

**DOI:** 10.1186/s40510-024-00536-0

**Published:** 2024-09-23

**Authors:** Yao Chen, Li Mei, Yuran Qian, Xinlianyi Zhou, Zhihe Zhao, Wei Zheng, Yu Li

**Affiliations:** 1 State Key Laboratory of Oral Diseases, National Center for Stomatology, National Clinical Research Center for Oral Diseases, Department of Orthodontics, West China Hospital of Stomatology, Sichuan University, Chengdu, 610041 China; 2 Discipline of Orthodontics, Department of Oral Sciences, Faculty of Dentistry, University of Otago, Dunedin, 9016 New Zealand; 3 State Key Laboratory of Oral Diseases, National Center for Stomatology, National Clinical Research Center for Oral Diseases, Department of Oral and Maxillofacial Surgery, West China Hospital of Stomatology, Sichuan University, Chengdu, 610041 China

**Keywords:** Orthodontic tooth movement, Gingival crevicular fluid, Biomarker, Cytokine, Bioinformatic analysis

## Abstract

**Background:**

The biological mechanisms driving orthodontic tooth movement (OTM) remain incompletely understood. Gingival crevicular fluid (GCF) is an important indicator of the periodontal bioprocess, providing valuable cues for probing the molecular mechanisms of OTM.

**Methods:**

A rigorous review of the clinical studies over the past decade was conducted after registering the protocol with PROSPERO and adhering to inclusion criteria comprising human subjects, specified force magnitudes and force application modes. The thorough screening investigated differentially expressed proteins (DEPs) in GCF associated with OTM. Protein-protein interaction (PPI) analysis was carried out using the STRING database, followed by further refinement through Cytoscape to isolate top hub proteins.

**Results:**

A comprehensive summarization of the OTM-related GCF studies was conducted, followed by an in-depth exploration of biomarkers within the GCF. We identified 13 DEPs, including ALP, IL-1β, IL-6, Leptin, MMP-1, MMP-3, MMP-8, MMP-9, PGE_2_, TGF-β1, TNF-α, OPG, RANKL. Bioinformatic analysis spotlighted the top 10 hub proteins and their interactions involved in OTM. Based on these findings, we have proposed a hypothetic diagram for the time-course bioprocess in OTM, which involves three phases containing sequential cellular and molecular components and their interplay network.

**Conclusions:**

This work has further improved our understanding to the bioprocess of OTM, suggesting biomarkers as potential modulating targets to enhance OTM, mitigate adverse effects and support real-time monitoring and personalized orthodontic cycles.

**Supplementary Information:**

The online version contains supplementary material available at 10.1186/s40510-024-00536-0.

## Introduction

Orthodontic treatment aims to move misaligned teeth into proper place by stimulating alveolar bone resorption and formation with orthodontic force. The biomechanical and molecular principle underpinning orthodontic tooth movement (OTM) is associated with expression of numerous regulatory molecules and critical to initiate the process of bone remodeling, leading to effective tooth movement. Understanding to the biomechanism in OTM is of great importance, since it may help improve the treatment efficiency and reduce the adverse side effects.

Periodontal ligament (PDL) plays a pivotal role in OTM [1]. Numerous animal studies [2–4] have investigated the molecular alterations in PDL in various OTM models. In clinical studies, immunostaining of the PDL is unapplicable; occasionally, premolars were stressed with rubber band [2] or buccolingually moved [5] followed by extraction of the teeth and harvest of the PDL for molecular assay. Nevertheless, such approaches may not represent the typical forms of OTM and could only observe responses at the initial OTM stage.

On the other hand, gingival crevicular fluid (GCF) serves as an effective indicator to monitor the time-course molecular expressions in periodontium. GCF is relatively independent and identifies specific biomarkers associated with several biological events with reasonable sensitivity [6–9]. Due to its similarity to serum, fluctuations in certain components of GCF may be utilized for the diagnosis or assessment of systemic diseases. Additionally, as GCF is derived from interstitial fluids within periodontal tissues, it holds promise as an oral biological fluid for detecting periodontal diseases like gingivitis [10, 11], periodontitis [12], caries [13], and external root resorption [14].

Originating from the microvascular system, GCF primarily comprises serum-borne molecular mediators, establishing itself as a suitable index for assessing activities during OTM [15]. This particular biofluid not only mirrors individual periodontal reactions to orthodontic forces, but also serves as an important measure for evaluating individual pubertal growth peak for growing subjects [16]. Owing to its non-invasive extraction and the practicality of consistent sampling from identical sites, mapping GCF biomarkers offers a substantial advantage for routine surveillance in OTM. This approach harbors the potential to catalyze the development of a predictive, preventive, and highly personalized paradigm in orthodontic care.

Data serve as a crucial asset for contemporary societies, poised to elevate healthcare’s quality, accessibility, cost-effectiveness, safety, and fairness. The realm of dental care and research is evolving into an era defined as data dentistry [17, 18]. The concept of “data dentistry” encapsulates the integration of Big Data and advanced data analytics, including artificial intelligence (AI) and machine learning, into dental, oral, and craniofacial (DOC) health care and research. This integration aims to deepen our understanding of patient health and disease, leading to more effective, efficient, and safer care. Bioinformatics is the research of biological abstracting in the context of macromolecules, as well as the use of informatics to organize the evidence acquired from these molecules and comprehend the derivations on a broader scale for use in multiple aspects of biology [19]. In the field of orthodontics, bioinformatics has tremendous potential to generate new perspectives between basic research and medical or dental applied sciences. However, current bioinformatics studies have predominantly focused on in vitro analyses [2, 20–22], with a significant emphasis on periodontal ligament cells (PDLCs). These studies aim to elucidate the gene regulatory mechanisms and pathway modifications implicated in OTM, yet they often yield evidence of a comparatively lower tier. Conversely, in vivo research has largely relied on animal models [23], such as rats and mice, highlighting a notable deficiency in studies utilizing human samples. A limited number of investigations have applied high-throughput sequencing to premolars extracted after the application of orthodontic force [2, 24], aiming to dissect the molecular underpinnings of OTM. However, these studies are generally constrained to examining OTM alterations at a singular time point due to sample limitations. Addressing this gap, this present study capitalizes on the benefits of GCF sampling. This approach enables a thorough investigation of OTM dynamics across multiple time points, amalgamating existing GCF-based OTM research with bioinformatics analysis. This strategy facilitates a dynamic landscape of the molecular evolution throughout the OTM process, aiming to deepen the understanding and offer novel insights into the biomechanisms of OTM.

## Methods

### Information sources

A critical review of the literature was undertaken following the registration of the protocol with PROSPERO (CRD42024529068). To identify all relevant studies, a systematic search was conducted across the following four databases: MEDLINE (via PubMed), Embase, Ovid, and Web of Science. Additionally, a supplemental manual search of the reference lists from related articles was performed, the details of which are provided in Supplementary Fig. 1. All searches were conducted in August 2023, and the year of publication was restricted as from 2013 to 2023.

### Eligibility criteria

The inclusion criteria used in this review were as follows: articles that related to all the items we listed in the review, including all the human GCF studies, that reported the orthodontic force magnitudes and tooth movement patterns, published within the last decade (from January 2013 to August 2023).

### Search strategy

The search strategy included three keywords: “orthodontic”, “gingival crevicular fluid” and “tooth movement”. The keyword “orthodontic” was expanded to “orthodontics” and “orthodontically”, and the abbreviation “OTM” and “GCF” were also concerned. The search strategy was as follows: (OTM[Title/Abstract] OR ((tooth movement [Title/Abstract]) AND ((GCF [Title/Abstract]) OR (gingival crevicular fluid [Title/Abstract])) AND (((orthodontic [Title/Abstract]) OR (orthodontics [Title/Abstract])) OR (orthodontically [Title/Abstract]))), which was developed for MEDLINE and adapted for the other databases.

### QUADAS assessment

The QUADAS (Quality Assessment of Diagnostic Accuracy Studies)-2 tool was used to evaluate risk of bias (ROB) for each study. It was performed independently by two researchers and in discrepancy, consensus by referring to a third evaluator (Supplementary Table 2). Within this analysis, the applicability of two studies was classified as unclear. Of the 17 studies examined, 12 exhibited both a low ROB and high applicability. The remaining five studies presented an unclear ROB, yet maintained high applicability.

### Protein interaction network and hub-proteins prediction

Protein-protein interaction and co-expression networks for the encoding of mutual target proteins were evaluated using the STRING database (https://string-db.org). The Top 10 key nodes and sub-networks in the given network were filtered out and ranked by topological algorithm of degree with the CytoHubba application installed in the Cytoscape software (version 3.9.1) [25].

## Results

### Study selection

The initial literature search yielded 495 papers, from which the duplicates were removed, leaving 261 articles for further evaluation. Among these, 27 studies were animal study and 156 studies were considered irrelevant to topic and excluded from the analysis. After a thorough assessment of the full texts of the remaining articles, 33 studies were excluded for not meeting the predetermined eligibility criteria, 11 studies were eliminated due to unavailability of full-text, Subsequently, 34 studies remained (Supplementary Table 1). According to the canine distalization and orthodontic force of 150 g, the review finally included 17 studies (Table 1), and explanations for the exclusion of studies are available in the Supplementary Fig. 1. Two reviewers (Y.C. and YR.Q.) screened the titles and abstracts of the identified studies independently. Consensus was obtained by discussion and consultation with a third reviewer (L.M.) to resolve any disagreements during study selection and data extraction.

**Table 1:** Variation trends of mediators in GCF under 150 g force

No.	Mediators	Sample (Number)	Age (Years)	Force (Gram)	Sampling (Time points)	Force application modes	Changes	Peak	PMID	Reference
1	Leptin	25	16–20	150–200	6 h, 21 days	Canine distalization	Increase	6 h	35017951	Alaguselvaraj et al., 2021 [26]
2	IL-1β	12	18–28	150	0, 7, 14, 21, 28 days	Canine distalization	Increase	7 days	34182967	Zheng et al., 2021 [86]
	OPG						Decrease	28 days		
	RANKL						-	NS		
3	PGE2	42	18 ± 4.5	150	0, 1, 2, 7 days	Canine distalization	Increase	1 day	24325834	Shetty et al., 2013 [37]
4	RANKL	10	12–16	150	0, 2, 7, 30, 45 days	Canine distalization	Increase	2 days	24346335	Domínguez et al., 2015 [69]
	OPG						Decrease	7 days		
5	IL-1β	16	18–24	150	1 h, 1 day, 7 days, 30 days, 60 days	Canine distalization	Increase	1 day	31463260	Singh et al., 2019 [40]
				50			Increase	2 days		
6	TNF-a	10	10–21	150	0, 1 h, 28 days	Canine distalization	Increase	1 h	30214696	Padisar et al., 2018 [30]
	IL-6						Increase	1 h		
7	IL-6	11	14–25	150	0, 1, 2, 3, 4 months	Canine distalization	Increase	1 month	27680969	Yassaei et al., 2016 [47]
8	Leptin	27	13–20	150	0, 1, 7, 21 days	Canine distalization	Decrease	21 days	31056072	Sar et al., 2019 [27]
9	MMP-1	16	13–27	150	-7 days, 0, 1 h, 1 day, 7 days, 14 days, 21 days	Canine distalization	Increase	1 day	22989715	Canavarro et al., 2013 [58]
	MMP-2						-	NS		
	MMP-3						Increase	1 day		
	MMP-7						-	NS		
	MMP-8						-	NS		
	MMP-12						-	NS		
	MMP-13						-	NS		
10	ALP	10	15–20	150	0, 1, 7, 14, 21, 28 days	Canine distalization	Decrease	28 days	26283826	Jeyraj et al., 2015 [89]
11	TNF-a	15	11–16	150	0, 1 day, 28 days	Canine distalization	Increase	28 days	30321318	Afacan et al., 2019 [31]
	IL-1Ra						Increase	28 days		
	IL-12						-	NS		
	GCSF						-	NS		
	IFN-a						-	NS		
	HGF						-	NS		
	VEGF						-	NS		
12	ALP	12	16–20	150	0, 7, 14, 21 days	Canine distalization	Increase	14 days	25808378	AlSwafeeri et al., 2015 [92]
13	IL-1β	10	14–25	150	0, 3, 7, 28, 56 days	Canine distalization	Increase	56 days	30268264	Varella et al., 2018 [43]
14	MMP-9	10	14–24	150	0, 14, 90 days	Canine distalization	Increase	14 days	32081593	Jivrajani et al., 2020 [60]
15	ALP	30	17–30	150	0, 7, 14, 28 days	Canine distalization	Increase	14 days	35674571	Raghav et al., 2022 [93]
16	IL-1β	20	18–24	150	0, 7, 28 days	Canine distalization	Increase	7 days	33459765	Erdur et al., 2021 [59]
	MMP-8						Increase	7 days		
	OPG						Decrease	7 days		
	RANKL						Increase	7 days		
17	IL-1β	15	12–19	150	0, 1, 7, 14, 21 days	Canine distalization	Increase	1 day	28289894	Üretürk et al., 2017 [41]
	TGF-β						Increase	7 days		

### Dynamics of molecules in GCF

The results of the studies are tabulated and presented in Table 1. While Table 2 provides an overview of the sampling times (highlighted in “S”), trends in concentration changes, and the peaks of the 13 DEPs as observed in various studies.

**Table 2:** Timeline summary

Mediators	Timeline									Sampling (Time points)
	< 1d	1d	2d	3d	7d	14d	21d	28d	> 28d	
IL-1β		↑			S	S	S			0, 1, 7, 14, 21 days
	S	↑			S				S	1 h, 1 day, 7 days, 30 days, 60 days
					↑			S		0, 7, 28 days
					↑	S	S	S		0, 7, 14, 21, 28 days
				S	S			S	↑	0, 3, 7, 28, 56 days
IL-6	↑							S		0, 1 h, 28 days
									↑	0, 1, 2, 3, 4 months
TNF-a	↑							S		0, 1 h, 28 days
		S						↑		0, 1 day, 28 days
PGE2		↑	S		S					0, 1, 2, 7 days
Leptin	↑						S			6 h, 21 days
		S			S		↓			0, 1, 7, 21 days
MMP-1	S	↑			S	S	S			-7 days, 0, 1 h, 1 day, 7 days, 14 days, 21 days
MMP-3	S	↑			S	S	S			-7 days, 0, 1 h, 1 day, 7 days, 14 days, 21 days
MMP-8					↑			S		0, 7, 28 days
MMP-9						↑			S	0, 14, 90 days
RANKL			↑		S				S	0, 2, 7, 30, 45 days
					↑			S		0, 7, 28 days
OPG			S		↓				S	0, 2, 7, 30, 45 days
					↓			S		0, 7, 28 days
					S	S	S	↓		0, 7, 14, 21, 28 days
TGF-β		S			↑	S	S			0, 1, 7, 14, 21 days
ALP					S	↑	S			0, 7, 14, 21 days
					S	↑		S		0, 7, 14, 28 days
		S			S	S	S	↓		0, 1, 7, 14, 21, 28 days

***(“S” signifies sampling time points***,*** arrows denote the trends and peak locations of observed changes.)***

#### Study characteristics

All 17 studies conducted were longitudinal in design, featuring sample collection at multiple time points. Baseline levels of all cytokines were used as internal controls for comparison. Each of these studies followed a standardized protocol for canine retraction, involving premolar extraction and the application of a 150 g orthodontic force. Importantly, all studies focused on analyzing modulators in gingival crevicular fluid (GCF).

#### Changing patterns of molecules

**Fig. 1:**
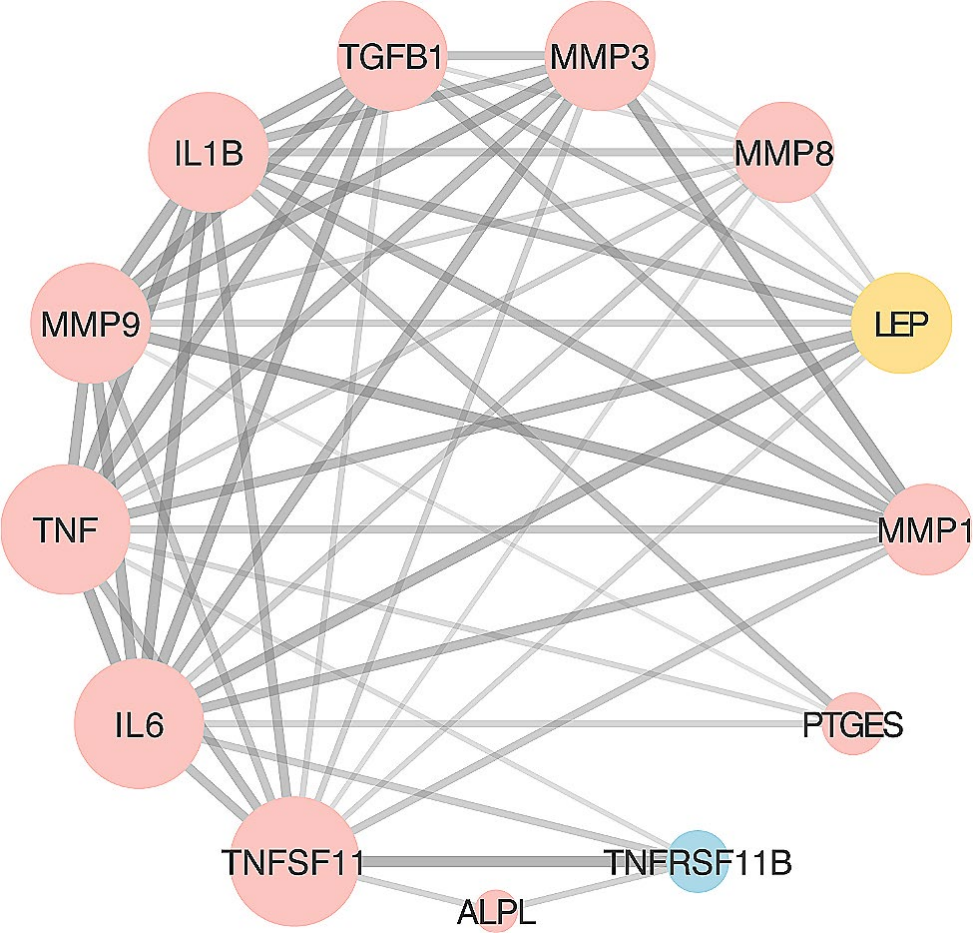
Potential inter-relationship among DEPs from the STRING database: (1) Node color is correlated with the concentration changes of the protein during the OTM process, blue (decrease), red (increase), biphasic (yellow). (2) Node size is positively correlated with the number of protein nodes interacting with it. (3) Edge thickness between nodes is positively correlated with the strength of the interaction between the two proteins

The expression profile of molecules in GCF showed variations at different observation times. Based on the above summarization about potential biomarkers, all proteins with statistically significant changes in expression levels compared to control groups during the OTM process are regarded as DEPs. We consistently identified 13 DEPs, including ALP, IL-1β, IL-6, Leptin, MMP-1, MMP-3, MMP-8, MMP-9, PGE2, TGF-β1, TNF-α, OPG, RANKL, listing in Fig. 1. Specifically, ALP (ALPL), IL-1β (IL1B), IL6, MMP1, MMP3, MMP8, MMP9, PGE_2_ (PTGES), TGF-β (TGFB1), and TNF-α (TNF) exhibited an upregulated trend in GCF during OTM. Conversely, OPG (TNFRSF11B) showed a downregulated trend during this process. Additionally, Leptin (LEP) displayed a biphasic fluctuation in GCF concentration during OTM [26, 27], potentially influenced by a range of factors including the applied force, duration of treatment, and individual variables such as obesity [28].

#### Interregulatory network prediction

Clinical orthodontics, being a complex biochemical phenomenon, involves various cytokines that interact to form a sophisticated network. This network regulates both hard tissue and soft tissue remodeling, presenting a challenge in summarization and interpretation. Consequently, we utilized bioinformatics as an effective tool to more comprehensively illustrate the intricate interactions among these 13 key hub-proteins.

**Fig. 2:**
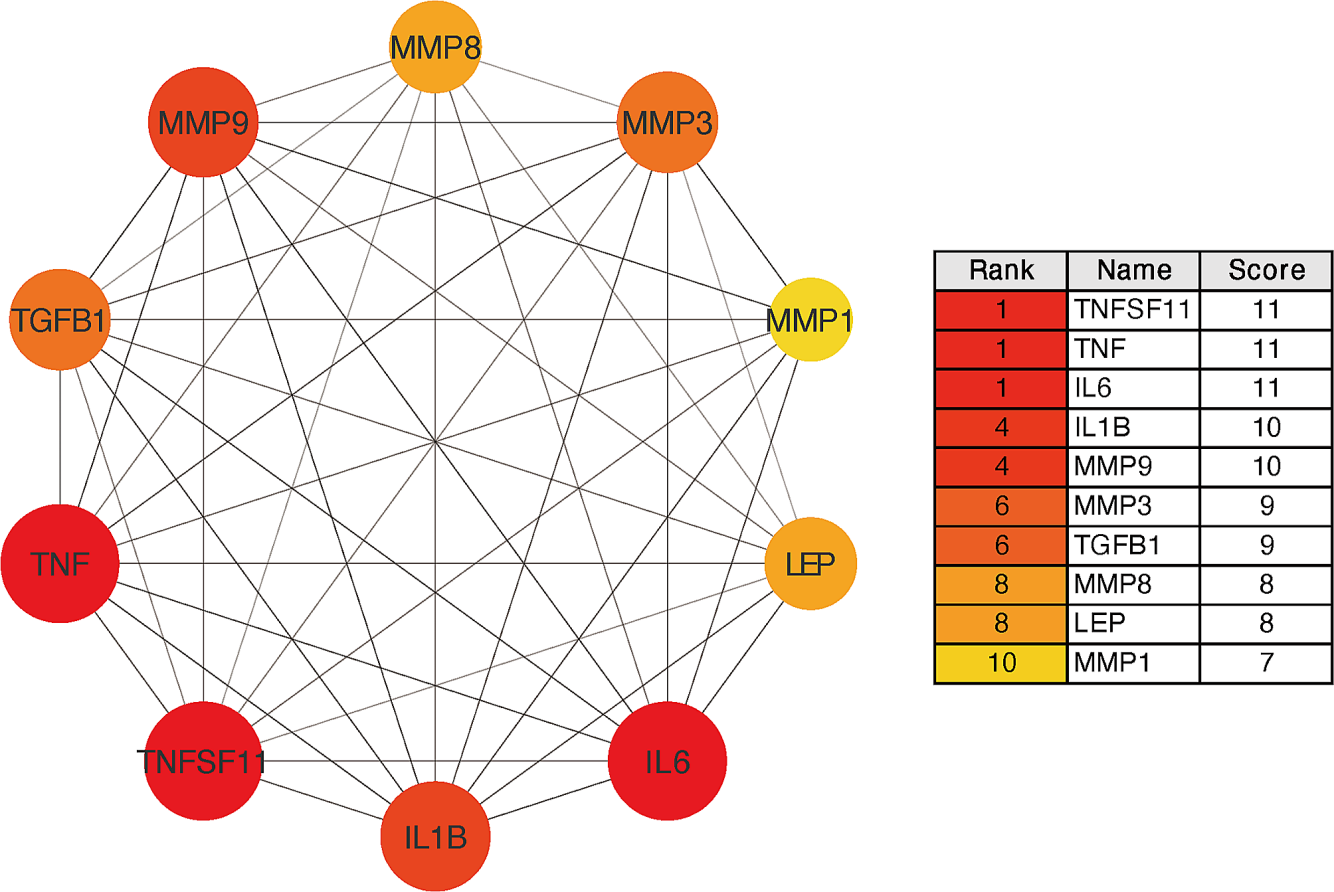
Top 10 key nodes and sub-networks ranked by topological algorithm of degree

PPI, as deciphered from the STRING database, revealed 52 notable interactions that serve as a foundation for understanding underlying mechanisms and identifying hub proteins. In Fig. 1, the node diameter indicates the number of interacting protein nodes. Node colors represent the protein’s concentration trend in GCF during the OTM process, with red signifying upregulation, blue indicating downregulation and yellow representing biphasic regulation. Furthermore, the edge thickness between nodes denotes the data’s reliability; a thicker line implies a more dependable and stronger interaction. Hub proteins defined with elevated connectivity are of particular significance. Their high connectivity scores underscore their importance. CytoHubba identified the top 10 nodes ranked by degree as hub proteins, specified as TNFSF11 (RANKL), TNF (TNF-α), IL6, IL1B (IL-1β), MMP9, MMP3, TGFB1 (TGF-β1), MMP8, LEP (Leptin), MMP1. The interactions and the ranking of these key protein nodes are depicted in Fig. 2, where a darker color indicates a protein’s greater importance within the interaction network.

## Discussion

### Latest potential biomarkers of OTM in GCF

Under the influence of orthodontic forces, PDL undergoes continuous physiological renewal. Although numerous in vivo and in vitro studies have explored the changes in various molecules during tooth movement, the results of all these studies exhibit significant discrepancies. Therefore, we have compiled a comprehensive summary of human GCF studies that explicitly indicate force magnitudes and tooth movement patterns from researches published in the last decade. This summary, presented in Supplementary Table 1, evaluates the variations and peak detections of all significant biomarkers in GCF during OTM. The table encompasses various parameters, including the number of participants, age groups, orthodontic force magnitudes, methods of force application, sampling time points, as well as trends and peak concentrations of potential biomarkers. This might enable a clearer understanding of the roles that different molecules play during this force-related periodontal bioprocess.

### Evaluating specific biomarkers in a time-dependent manner

The cytokine network plays a crucial role in the metabolic transition of bone remodeling. This includes the promotion of proinflammatory cells by cytokines, the modulation of osteoblast proliferation and differentiation by growth factors, and the regulation of osteoclasts by bone resorption-promoting factors. Reflecting on the main functions and concentration changes of each regulatory factor in GCF-based clinical studies, we categorized the OTM process into three phases. Integrating this framework with the temporal changes of mediators observed in the OTM process, along with the results from the above PPI analysis, enables us to construct a GCF-based time course landscape of OTM.

#### Phase 1: 0 ∼ 24 h

When subjected to continuous orthodontic force, the teeth cause bending and deformation of the alveolar bone under stress, leading to the drainage of periodontal tissue fluid and initiating the early stages of tooth tipping within the bony socket [29]. This mechanotransduction process modifies the periodontal microenvironment through changes in oxygen pressure and metabolic signals, sparking a cascade of cellular and molecular activities crucial for orthodontic tooth movement. During this initial phase, there is a significant synthesis and secretion of various proinflammatory enzymes, cytokines, and chemokines.

The primary response to mechanical stress is the secretion of tumor necrosis factor-alpha (TNF-α), reaching its maximal levels one hour after force application [30]. Whereas, another randomized controlled trial, constrained by sampling time points (limited to day 1 and day 28), indicated a rise in TNF-α levels by the latter date [31]. Previous study has reported that TNF-α stimulation promotes bone resorption and inhibit bone formation, making it a significant indicator of osteoclast activity [32]. Upon binding to its receptor, TNF-α induces RANK expression in osteoclast precursors [33], and triggers induction of the Wnt signaling inhibitor Dkk1 in the osteoblasts at the mRNA and protein levels, with a simultaneous increase in the receptor activator of NF-κB ligand (RANKL) [34]. Furthermore, it enhances the expression of sclerostin [35], which coincides with the upregulation of RANKL [36], thereby further promoting osteoclastogenesis during OTM. Prostaglandin E2 (PGE_2_), another critical mediator of inflammation, significantly amplifies the inflammatory response. It promotes the secretion and synthesis of interleukins, peaking in concentration within 24 h [37]. Previous research has identified a relationship between these two mediators: TNF-α can increase RANKL expression through PGE_2_-induced activation of NFATc1 [38], which is in accordance with the trend of above GCF-based clinical results. Both submucosal and intraligamentous PGE_2_ administration significantly enhanced the rate of tooth movement and bone metabolism [39], indicating a potential therapeutic usage of PGE_2_ in accelerating OTM process. As pain is among the most cited negative effects of orthodontic treatment, a human study confirmed that acetaminophen showed no significant effect on PGE_2_ synthesis and may be the safe choice compared to ibuprofen for relieving pain associated with OTM [37].

Whilst, interleukins are activated, being essential in recruiting neutrophils, leukocytes, and other inflammatory mediators. Previous findings have shown that IL-1β is released within the first hour immediately following the application of orthodontic force, and reaches its peak within 24 h [40–42]. In a split-mouth clinical trial of Low-Level Laser Therapy (LLLT), IL-1β in the control side peaked at day 56, possibly due to the relative impact of therapeutic interventions applied on the experimental counterpart [43]. In response to mechanical stress, IL-1β enhances bone resorption by activating osteoclasts and attracting leukocytes and other inflammatory agents that contribute to bone remodeling [44]. As one of the most abundant inflammatory mediators, IL-1β increased MMP-1 synthesis and MMP-2 gene expression and decreased TIMP-1 gene expression in the presence of in vitro cyclic low-magnitude orthodontic tensile forces [45]. IL-6, featured as one of the top 10 DEPs, serves as a multidirectional cytokine and a key regulator of bone resorption. The concentration of IL-6 in GCF shows an increase within one hour post-application of stress and peaks at 24 h [30, 46]. In another clinical study with a broader sampling timeframe (monthly collections over a continuous four-month period), it was observed that IL-6 concentrations peaked in the first month of orthodontic therapy, implicating a critical role for IL-6 in the early stage of orthodontic treatment processes [47]. Earlier research has proposed that IL-6 can amplify the effects of IL-1β and TNF-α, enhancing their biological impacts and promoting osteoclastogenesis [48]. After orthodontic intervention, IL-6 signal was activated and found to increase the number of osteoclasts by suppressing apoptosis and increasing their responsiveness to macrophage colony-stimulating factor (M-CSF) and RANKL [49]. Another research conducted that IL-6 increased osteocyte-mediated osteoclastic differentiation by activating RANKL and JAK2 [50]. IL-6 activates STAT3 via glycoprotein 130 (gp130) [51] to stimulate osteoclast progenitors proliferation, leading to bone resorption and in concert with other bone-resorbing agents at the early stage of OTM [52, 53]. Synthesizing the conclusions of the aforementioned studies, it becomes evident and clear that the IL-6/GP130/JAK2/STAT3 signaling axis is fundamentally significant during the first phase, leading to the initiation of upcoming tooth movement progression.

Our findings from the initial phase of tooth movement (1 month of tooth alignment) corroborate those of other studies [54, 55]. Building upon the above discussed potential mechanisms, this phase is characterized by the synthesis of proinflammatory cytokines and osteoclast differentiation-inducing molecules at the level of secreted proteins, setting the stage for the tooth movement process to transition to the subsequent phase.

#### Phase 2: 24 h ∼ 7d

In this stage of tooth movement, the primary focus is on the remodeling of the periodontal ligament (PDL). The PDL is a unique connective tissue comprising a heterogenous cell population (PDLCs) and a fibrous extracellular matrix (ECM) [56]. Under the influence of orthodontic forces, PDL cells secrete matrix proteins that contribute to ECM deposition. Concurrently, ECM degradation occurs through the expression of various proteolytic enzymes, including matrix metalloproteinases (MMPs) and their specific inhibitors, the tissue inhibitors of metalloproteinases (TIMPs), function in a coordinated manner to regulate the remodeling of periodontal tissue [57].

Under a specific orthodontic load of 150 g, the concentrations of various subtypes of MMPs undergo distinct changes. MMP-1 (collagenase) and MMP-3 (stromelysin) are observed to increase significantly on the first day of OTM [58]. MMP-8, another collagenase, is elevated to its highest concentration on day 7 [59]. Meanwhile, MMP-9, functioning as a gelatinase, shows its concentration peak on day 14. However, this observed peak is actually constrained by the limited sampling time points in the study’s GCF collection, which were only on day 0, day 14, and day 90 [60]. Therefore, it exhibits a relatively slower rise compared to other MMPs. Other studies with different force values have shown a significant increase in its concentration at earlier day 7 [61, 62].

In vitro experiments show that IL-1β significantly boosts MMP-1 production in human Periodontal Ligament Mesenchymal Stem Cells (hPDL-MSCs) in a time-dependent manner [45]. Mechanical force is capable of stimulating MMP-3 expression, potentially via the p38 MAPK pathway, with the most pronounced signaling observed at 24 h. This mechanical responsiveness within MMP-3 promoter regions has been noted in both human and animal models, as shown in both in vitro and in vivo studies [63]. In human PDL cells, MMP3 expression is enhanced by IL-6 upregulated through cyclic tensile force, contributing to the maintenance of periodontal homeostasis [64]. When cultured PDL cells are exposed to TNF-α and analyzed with an MMP antibody array, MMP-3 is identified as the most significantly upregulated protein [65]. TNF-α also enhances MMP-9 expression mediated through the NF-κB element in MMP-9 promoter, leading to the release of soluble intercellular adhesion molecule-1 [66]. Moreover, a significant correlation is observed between Lipocalin-2/matrix metalloproteinase 9 (MMP9/NGAL) and Thrombospondin-1 (TSP1), indicating their heightened involvement in the angiogenesis within the PDL during orthodontic periodontal remodeling [67]. The loss of TIMP activates the Metalloproteinase-TNFα-DKK1 axis, undermining Wnt signaling and leading to a reduction in bone mass [34].

Through MMP activity, osteoid can be degraded by osteoblasts so that the differentiated osteoclasts could attach to the bone surface before the actual bone resorption. Classical bone remodeling markers like RANKL exhibit fluctuations in this phase. A randomized clinical trial indicates that the upregulation of RANKL and downregulation of OPG both peak on day 7 [68]. Based on other established clinical GCF results, the concentration of RANKL is also significantly upregulated during this phase [69, 70].

The elevation of RANKL facilitates its binding to RANK on the surface of osteoclasts precursors (OCPs), which in turn promotes the differentiation of OCPs into osteoclasts. This differentiation leads to enhanced bone resorption activity under the influence of osteoclasts.

Leptin, known to orchestrate the host’s response to inflammatory and infectious stimuli, has been shown to undergo a biphasic change in GCF levels during OTM, underscoring its substantial link with the rate of tooth movement. Alaguselvaraj et al. reported an elevation in GCF leptin concentrations shortly after the application of orthodontic force [26]. Nonetheless, the temporal resolution of their observations, confined to only 6 h and 21 days post-application, which does not comprehensively delineate the dynamic timeline of leptin concentration changes. Contrastingly, an in vivo clinical investigation with an expanded array of sampling intervals revealed a marked decline in leptin concentrations, plummeting from baseline values to 21 days (*P* = 0.0001) during the process of canine retraction [27]. In other leptin-related investigations, where specific force magnitudes were not specified, leptin concentrations initially increased within the first 24 h, followed by a decline and then a rise to levels proximate to baseline [71, 72]. This biphasic change pattern intimates a robust association between fluctuations in leptin concentration and the dynamics of tooth movement.

Leptin assumes a multifaceted role in the dynamics of OTM, mediating its effects through an array of cellular and molecular interactions. It provokes the secretion of pro-inflammatory cytokines IL-6 and IL-8 in human periodontal ligament cells (hPDLCs) via binding to the obesity-related leptin and leptin receptor b (OBRb), thereby triggering subsequent intracellular signaling cascades [73]. Moreover, leptin markedly attenuates the levels of growth factors (TGFβ1, VEGFA), transcription factors (RUNX2), matrix molecules (collagen, periostin), while concurrently suppressing SMAD signaling pathways during regenerative processes [74]. Intriguingly, administration of leptin, either in vivo and in vitro, inhibits the expression of RANKL. This suppression extends to the mechanical force-induced up-regulation of RANKL in hPDLCs, which was rescued by LepR siRNA transfection [75]. Furthermore, leptin exacerbates the response of cementoblasts to compressive forces, escalating PGE2 secretion and apoptotic activity, and thus, increased levels of leptin may influence the inflammatory response during orthodontically induced tooth movement [76]. This complex interplay of leptin with various cellular and molecular mechanisms underscores its significant yet intricate role in the regulation of OTM.

#### Phase 3: 7d ∼ 30d

Following bone resorption, a variety of cells including monocytes, osteocytes and pre-osteoblasts are recruited to begin bone formation. One of the coupling signals linking of bone turnover is bone matrix-derived transforming growth factor beta (TGF-β) [77], playing a major role in bone and cartilage mechanobiological signaling [78]. During phase 3, the absolute TGF-β1 level was detected a significant increase after force application [70, 79]. Recent advances in cellular mechanobiology highlight a feedback loop links TGFβ signaling and ECM material quality via cytoskeletal tension [78, 80]. Osteoclasts release and activate TGFβ stored in latent form in the bone matrix during resorption through creating an acidic microenvironment, as well as through the secretion of matrix metalloproteinases [81]. MMP2 and MMP9 were able to cleave latent TGF-β-binding protein-1 (LTBP1) to release TGF-β from ECM-bound stores [82], potentially the first step in the pathway by which matrix-bound TGF-β is rendered active. Since then, it has been reported that TGF-β inhibited the production of RANKL by osteoblasts thus decreasing osteoclastic resorption [83, 84]. On the other hand, TGF-β also enhances early osteoblast differentiation by encouraging the recruitment and proliferation of osteoblast precursors, as well as the expression of their product proteins [85].

Osteoclasts are now replaced by osteoblast-lineage cells which initiate bone formation. Concurrently, osteoprotegerin (OPG), synthesized by osteoblasts, acts as a decoy receptor for RANKL, effectively inhibiting the RANK-RANKL interaction. Following this, OPG concentration diminishes as a response to RANKL’s elevation, crucial for maintaining the balance of bone remodeling. Clinical evidence demonstrates a rapid decline in OPG concentration by day 7, followed by a slight increase by day 21, and then a subsequent decrease to the lowest level by day 28 [86]. Since then, high ALP activity was detected in the osteoid areas of new bone formation [87] and its GCF concentration elevated significantly at days 14 to facilitate mineralization process [88, 92, 93]. Conversely, alternate research delineates a significant reduction in ALP levels subsequent to force application, with a progressive declining trend in ALP activity observed on both the distal aspect of the canine and the mesial aspect of the second premolar [89]. Despite that, ALP is involved in bone mineralization, where its primary role is to catalyze the hydrolysis of monophosphate esters at high pH [90]. Its expression and activity are key markers of osteoblast differentiation and maturation [91].

**Fig. 3:**
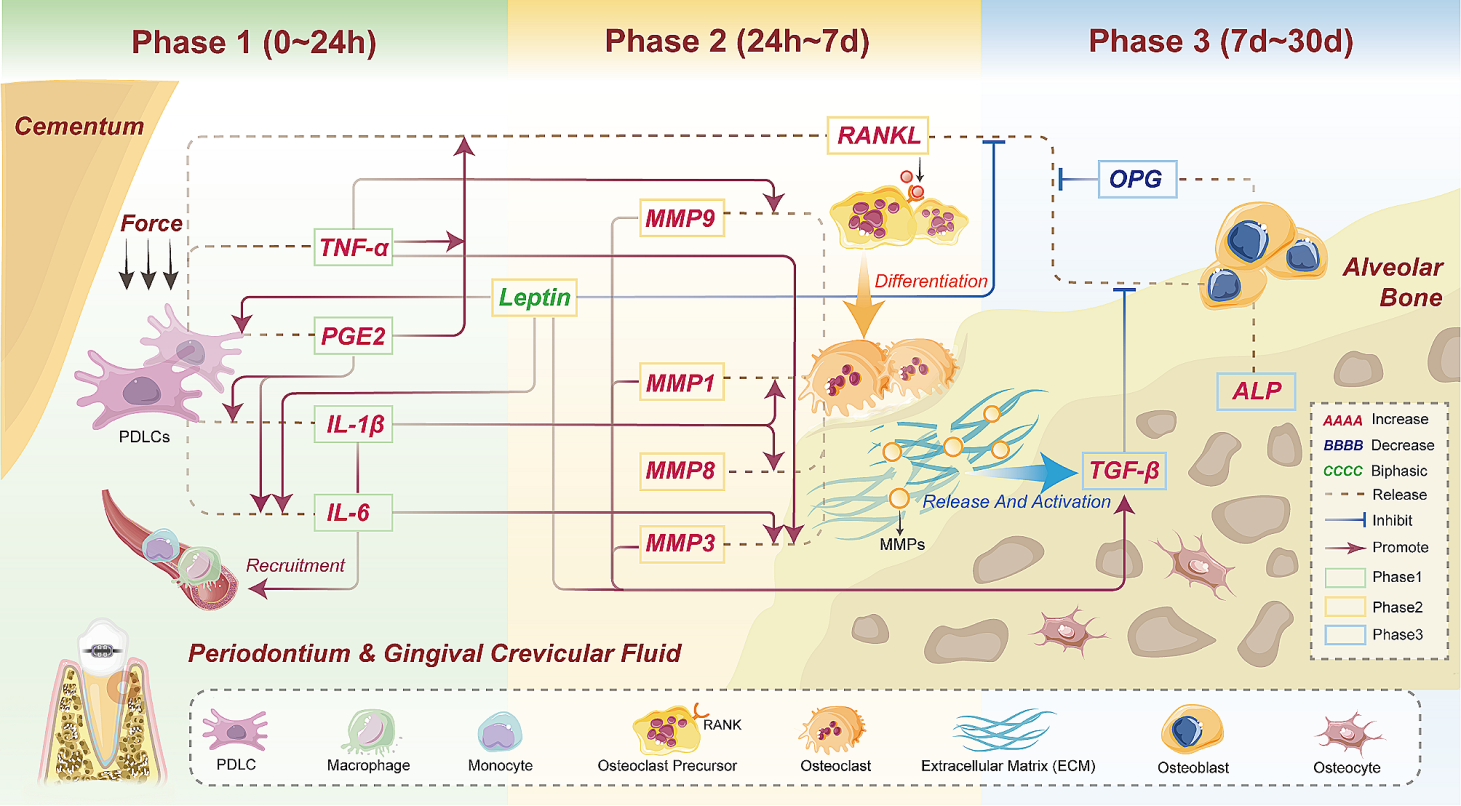
Hypothetical OTM mechanisms in a time-dependent manner

All the potential molecular mechanisms identified are in line with trends observed in the expression levels of clinical GCF and PPI results. Based on these findings, we have integrated big data and both in vitro and in vivo experimental evidence to construct a network interaction hypothesis diagram for 13 DEPs across the three phases of OTM, as illustrated in Fig. 3.

## Conclusions

1. Multiple secretory proteins have been studied in human GCF, which may serve as biomarkers for tooth movement. These include ALP (ALPL), IL-1β (IL1B), IL6, MMP1, MMP3, MMP8, MMP9, PGE_2_ (PTGES), TGF-β (TGFB1), and TNF-α (TNF), aiming to elucidate the orthodontic bioprocess.
2. Bioinformatic analysis spotlighted the top 10 hub proteins, including TNFSF11, TNF, IL6, IL1B, MMP9, MMP3, TGFB1, MMP8, LEP, MMP1 and their interactions involved in OTM. These key genes offer potential for precise modulation in future orthodontic interventions, positioning them as promising candidates to accelerate tooth movement while minimizing adverse effects.
3. A hypothetic diagram was proposed for the time-course bioprocess in OTM, which involves three phases containing specific cellular and molecular components, biomechanical events and their interplay network.

### Limitations

Proteins can be classified into membrane-bound and secretory categories. This review predominantly focuses on secretory proteins within the GCF, thus presenting limitations in the scope of detection targets. The cellular origin of these proteins is challenging to ascertain with precision, necessitating reliance on speculations based on prior research findings. The studies included in this review selectively focused on quantitative analysis of potential biomarkers, reflecting the subjective inclinations of researchers, which, although scientifically valid, may bias the selection of study targets. This subjectivity highlights the importance of integrating high-throughput molecular detection techniques like proteomics to achieve a more comprehensive and objective overview of OTM’s underlying mechanisms. Moreover, the scope of the reviewed studies is often limited to specific populations and time points, and constrained by the number of available publications. Consequently, the derived conclusions are based on assumptions and theories grounded in the data, potentially limiting their applicability across diverse populations or in different circumstances.

## Electronic supplementary material

Below is the link to the electronic supplementary material.

**Supplementary Material 1**: **Supplementary Fig. 1** PRISMA flowchart.

**Supplementary Material 2**: **Supplementary Table 1.** Mediators from all the human GCF studies which were clearly marked force magnitudes and tooth movement patterns in the last decade.

**Supplementary Material 3**: **Supplementary Table 2.** QUADAS assessment.

**Supplementary Material 4**: **Supplementary Table 3.** Rankings of DEGs.

**Supplementary Material 5**: **Supplementary Table 4.** STRING interactions.

**Supplementary Material 6**: **Supplementary Table 5** STRING mapping.

## Data Availability

Not applicable.

## Abbreviations

OTM: Orthodontic tooth movement
GCF: Gingival crevicular fluid
DEP: Differentially expressed protein
PPI: Protein-protein interaction
AI: Artificial intelligence
PDL: Periodontal ligament
PDLC: Periodontal ligament cell
QUADAS: Quality Assessment of Diagnostic Accuracy Studies
ROB: Risk of bias
LLLT: Low-level laser therapy
ECM: Extracellular matrix
TIMP: Tissue inhibitors of metalloproteinase
MMP: Matrix metalloproteinase
OCP: Osteoclast precursor
ALP: Alkaline, IL-1β, IL-6
PGE_2_: Prostaglandin E2
TGF-β: Transforming growth factor beta
TNF-α: Tumor necrosis factor-alpha
OPG: Osteoprotegerin
RANKL: Receptor activator of NF-κB ligand
