# Supplementary figures and images for: Integrated bioinformatic analysis of protein landscape in gingival crevicular fluid unveils sequential bioprocess in orthodontic tooth movement
Source: Prog Orthod. 2024 Sep 23;25:37. doi: 10.1186/s40510-024-00536-0 (PMC11417088; doi:10.1186/s40510-024-00536-0)

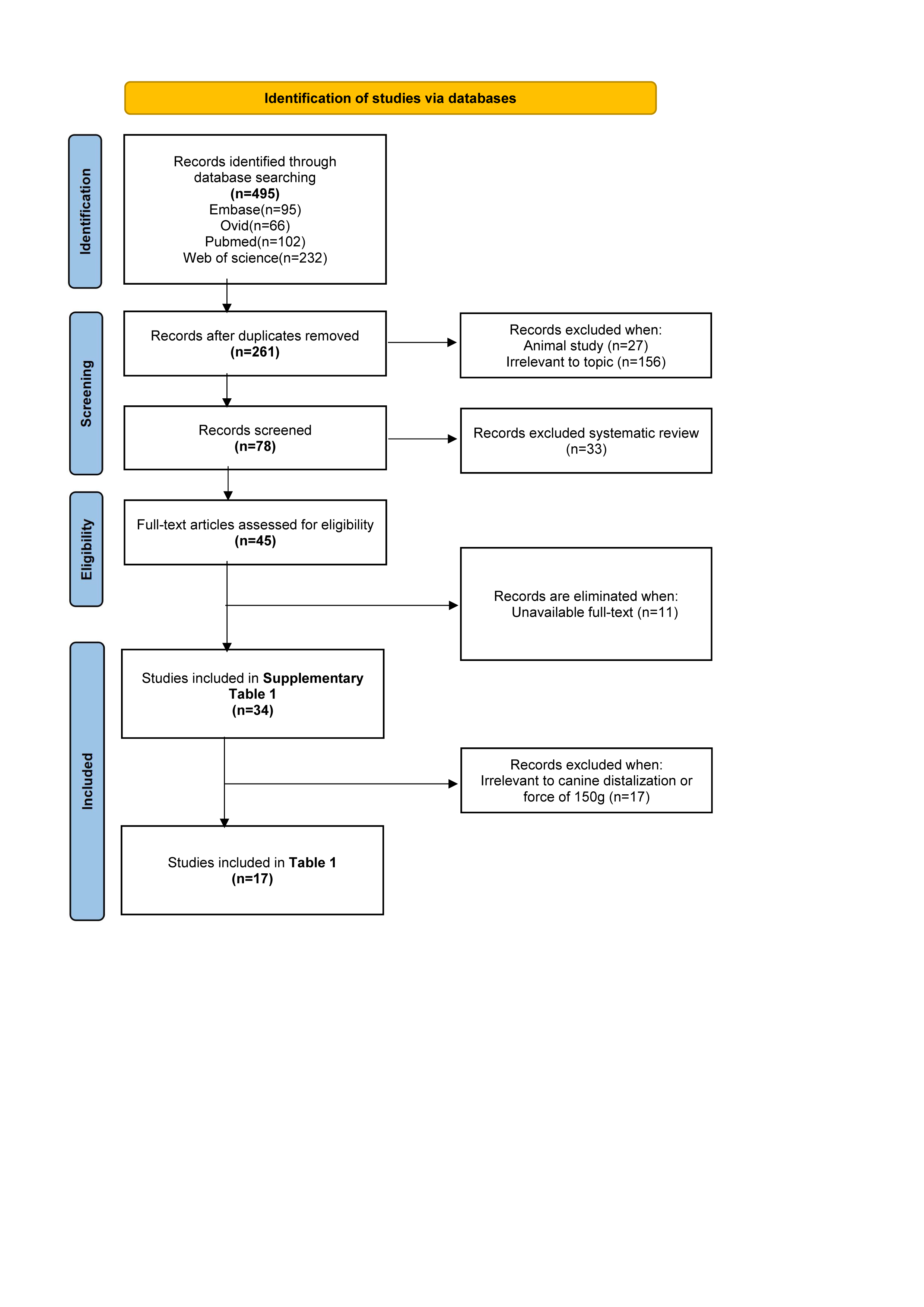

Supplement: Supplementary file 1 — Supplementary Material 1: Supplementary Fig. 1 PRISMA flowchart. [file 40510_2024_536_MOESM1_ESM.tif]
