# Supplementary material for: Integrated bioinformatic analysis of protein landscape in gingival crevicular fluid unveils sequential bioprocess in orthodontic tooth movement
Source: Prog Orthod. 2024 Sep 23;25:37. doi: 10.1186/s40510-024-00536-0 (PMC11417088; doi:10.1186/s40510-024-00536-0)
Supplement: Supplementary file 2 — Supplementary Material 2: Supplementary Table 1. Mediators from all the human GCF studies which were clearly marked force magnitudes and tooth movement patterns in the last decade. [file 40510_2024_536_MOESM2_ESM.docx]

***Supplementary Table 1. Mediators from all the human GCF studies which were clearly marked force magnitudes and tooth movement patterns in the last decade***

| **No.** | **Mediators** | **Sample (Number)** | **Age (Years)** | **Force (Gram)** | **Sampling**  **(Time points)** | **Force application modes** | **Changes** | **Peak** | **PMID** |
| --- | --- | --- | --- | --- | --- | --- | --- | --- | --- |
| 1 | Leptin | 25 | 16-20 | 150 -200 | 6 hour, 21 days | Canine distalization | Increase | 6 hours | 35017951 |
| 2 | IL-1β | 12 | 18-28 | 150 | 0, 7, 14 days | Canine distalization | Increase | 7 days | 34182967 |
|  | OPG |  |  |  |  |  | Decrease | 28 days |  |
|  | RANKL |  |  |  |  |  | - | NS |  |
| 3 | PGE2 | 42 | 18±4.5 | 150 | 0, 1, 2, 7 day(s) | Canine distalization | Increase | 1 day | 24325834 |
| 4 | RANKL | 10 | 12-16 | 150 | 0, 2, 7, 30, 45 days | Canine distalization | Increase | 2 days | 24346335 |
|  | OPG |  |  |  |  |  | Decrease | 7 days |  |
| 5 | IL-1β | 21 | 12-20 | 100 | 4 hours, 7 days, 42 days | Canine distalization | Increase | 4 hours | 22987319 |
|  | IL-8 |  |  |  |  |  | Increase | 4 hours |  |
|  | TNF-a |  |  |  |  |  | Increase | 4 hours |  |
|  | MMP-9 |  |  |  |  |  | Increase | 7 days |  |
|  | TIMP1/2 |  |  |  |  |  | Increase | 7 days |  |
|  | RANKL |  |  |  |  |  | Increase | 42 days |  |
| 6 | IL-1β | 16 | 18-24 | 150 | 1 hour, 1, 7, 30, 60 day(s) | Canine distalization | Increase | 1 days | 31463260 |
|  |  |  |  | 50 |  |  | Increase | 2 days |  |
| 7 | TNF-a | 10 | 10-21 | 150 | 0, 1 hour, 28 days | Canine distalization | Increase | 1 hour | 30214696 |
|  | IL-6 |  |  |  |  |  | Increase | 1 hour |  |
| 8 | CEMP-1 | 11 | 18.3-37.7 | 100 | 0, 1, 7, 14, 28, 56 day(s) | Premolar diatalization | Increase | 56 days | 33822289 |
|  | DPP |  |  |  |  |  | Decrease | 7 days |  |
|  | CTX-1 |  |  |  |  |  | Increase | 56 days |  |
| 9 | IL-6 | 11 | 14-25 | 150 | 0, 1, 2, 3, 4 month(s) | Canine distalization | Increase | 1 month | 27680969 |
| 10 | Leptin | 27 | 13-20 | 150 | 0, 1, 7, 21 day(s) | Canine distalization | Decrease | 21 days | 31056072 |
| 11 | Calcitonin | 15 | 10-15 | 100 | 0, 1 hour, 1, 7, 15 day(s) | Upper midline diastema closure | Increase | 7 days | 23107048 |
| 12 | MMP-1 | 16 | 13-27 | 150 | -7 days, 0, 1 hour,  1, 7, 14, 21 day(s) | Canine distalization | Increase | 1 day | 22989715 |
|  | MMP-2 |  |  |  |  |  | - | NS |  |
|  | MMP-3 |  |  |  |  |  | Increase | 1 day |  |
|  | MMP-7 |  |  |  |  |  | - | NS |  |
|  | MMP-8 |  |  |  |  |  | - | NS |  |
|  | MMP-12 |  |  |  |  |  | - | NS |  |
|  | MMP-13 |  |  |  |  |  | - | NS |  |
| 13 | ALP | 10 | 15-20 | 150 | 0, 1, 7, 14, 21, 28 day(s) | Canine distalization | Decrease | 28 days | 26283826 |
| 14 | MMP-2 | 15 | 12-15 | 150 | 0, 7, 14, 21, 28, 32 days | Beta-titanium archwire ligation | - | NS | 32809076 |
|  | MMP-9 |  |  |  |  |  | - | NS |  |
| 15 | ALP | 12 | 11-17 | 250 | 0, 14, 28 days | Canine distalization | Increase | 14 days | 26535403 |
|  | ACP |  |  |  |  |  | Increase | 14 days |  |
| 16 | IL-1β | 10 | 15.6 ± 0.9 | 250 | 1 hour, 1 day, 7 days | Canine distalization | Increase | 1 day | 31198722 |
|  | IL-6 |  |  |  |  |  | Increase | 1 day |  |
|  | TNF-a |  |  |  |  |  | Increase | 1 day |  |
|  | β2-MG |  |  |  |  |  | Increase | 7 day |  |
|  | EGF |  |  |  |  |  | Increase | 1 day |  |
| 17 | miRNA-29 | 15 | 10-17 | 250 | 1 hour, 1, 7, 42 day(s) | Canine distalization | Increase | 42 days | 29518149 |
| 18 | miR-34a | 20 | 12-18 | 130 | 0, 1h, 1, 7, 28, 84 day(s) | Canine distalization | Decrease | 28 days | 33378474 |
|  | MMP-2 |  |  |  |  |  | Increase | 28 days |  |
|  | MMP-9 |  |  |  |  |  | Increase | 28 days |  |
|  | MMP-14 |  |  |  |  |  | Increase | 28 days |  |
| 19 | TGF-β | 10 | 22.3 ± 3.3 | 100 | 0, 1 hour, 7, 21 days | Aligning | Increase | 21 days | 27409364 |
|  | IL-1β |  |  |  |  |  | Increase | 21 days |  |
|  | RANKL |  |  |  |  |  | Increase | 21 days |  |
|  | OPG |  |  |  |  |  | Decrease | 7 days |  |
|  | OPN |  |  |  |  |  | Increase | 21 days |  |
| 20 | IL-1β | 15 | 19-25 | 60 | 0, 1, 2, 3 month(s) | Canine distalization | Increase | 3 month | 25811245 |
| 21 | NO | 20 | 17.8 ± 4.2 | 80 | 0, 2, 3, 5, 7, 9, 14, 16, 21, 23, 28, 30 days | Lateral Increaseisor distalization | - | NS | 22350425 |
| 22 | TNF-a | 15 | 11–16 | 150 | 0, 1, 28 day(s) | Canine distalization | Increase | 28 days | 30321318 |
|  | IL-1Ra |  |  |  |  |  | Increase | 28 days |  |
|  | IL-12 |  |  |  |  |  | - | NS |  |
|  | GCSF |  |  |  |  |  | - | NS |  |
|  | IFN-a |  |  |  |  |  | - | NS |  |
|  | HGF |  |  |  |  |  | - | NS |  |
|  | VEGF |  |  |  |  |  | - | NS |  |
| 23 | ALP | 12 | 16-20 | 150 | 0, 7, 14, 21 days | Canine distalization | Increase | 14 days | 25808378 |
| 24 | IL-1β | 10 | 14-25 | 150 | 0, 3, 7, 28, 56 days | Canine distalization | Increase | 56 days | 30268264 |
| 25 | MMP-9 | 10 | 14-24 | 150 | 0, 14, 90 days | Canine distalization | Increase | 14 days | 32081593 |
| 26 | ALP | 30 | 17-30 | 150 | 0, 7, 14, 28 days | Canine distalization | Increase | 14 days | 35674571 |
| 27 | IL-1β | 20 | 18-24 | 150 | 0, 7, 28 days | Canine distalization | Increase | 7 days | 33459765 |
|  | MMP-8 |  |  |  |  |  | Increase | 7 days |  |
|  | OPG |  |  |  |  |  | Decrease | 7 days |  |
|  | RANKL |  |  |  |  |  | Increase | 7 days |  |
| 28 | IL-1β | 15 | 12-19 | 150 | 0, 1, 7, 14, 21 day(s) | Canine distalization | Increase | 1 day | 28289894 |
|  | TGF-β |  |  |  |  |  | Increase | 7 days |  |
| 29 | IL-1β | 18 | 11-14  21-45 | 50 | 0, 1, 7, 14, 28 day(s) | Canine distalization | Increase | 1 day | 29706211 |
|  | CCL2 |  |  |  |  |  | Increase | 1 day |  |
|  | TNF-a |  |  |  |  |  | Increase | 1 day |  |
|  | RANKL |  |  |  |  |  | Increase | 7 days |  |
|  | MMP-9 |  |  |  |  |  | Increase | 1 day |  |
| 30 | Chondroitin Sulphate | 16 | 12.3–22.5 | 70 | 0, 1, 2, 3, 4, 5, 6, 7, 8 week(s) | Canine distalization | Increase | 1 week | 23535117 |
|  |  |  |  | 120 |  |  | Increase | 1 week |  |
| 31 | DPSP | 8 | 13-18 | 150 | 0, 7, 21, 28, 35 days | Buccally directed tipping force | Increase | 7 days | 34927213 |
| 32 | IL-6 | 20 | 35-45 | 150 | 1, 3, 7 day(s) | Molar intrusion | Increase | 3 days | 31001928 |
|  | IL-8 |  |  |  |  |  | Increase | 3 days |  |
|  | IL-1a |  |  |  |  |  | Increase | 3 days |  |
| 33 | IL-1β | 8 | 13.9–22.9 | 225 | 0, 3 hours, 1, 3, 7, 28 day(s) | TMA cantilever spring | Increase | 1 day | 28762151 |
|  | IL-4 |  |  |  |  |  | Increase | 1 day |  |
|  | IL-6 |  |  |  |  |  | - | NS |  |
|  | IL-7 |  |  |  |  |  | Increase | 28 days |  |
|  | IL-8 |  |  |  |  |  | - | NS |  |
|  | IFN-γ |  |  |  |  |  | Increase | 3 days |  |
|  | GM-CSF |  |  |  |  |  | Increase | 28 days |  |
|  | TNF-a |  |  |  |  |  | Increase | 28 days |  |
| 34 | TNF-a | 23 | 10-24 | 100 | 0, 1, 3, 7, 14, 21, 28 day(s) | Beta-titanium alloy cantilever | Increase | 3 days | 26232838 |
|  | IFN-γ |  |  |  |  |  | Increase | NS |  |
|  | IL-2 |  |  |  |  |  | Increase | NS |  |
|  | IL-4 |  |  |  |  |  | Increase | 3 days |  |
|  | IL-6 |  |  |  |  |  | Increase | NS |  |
|  | IL-10 |  |  |  |  |  | Increase | NS |  |
|  | IL-17A |  |  |  |  |  | Increase | 7 days |  |
